# Effect of line and floor type on growth performance and feather characterization during the growth period of White Roman geese

**DOI:** 10.5713/ajas.19.0663

**Published:** 2019-10-22

**Authors:** Min Jung Lin, Shen Chang Chang, Tzu Jou Chen, Wei Chih Lin, Shao Yu Peng, Tzu Tai Lee

**Affiliations:** 1 Changhua Animal Propagation Station, Livestock Research Institute, Council of Agriculture, Executive Yuan, Changhua 52149, Taiwan; 2 Kaohsiung Animal Propagation Station, Livestock Research Institute, Council of Agriculture, Executive Yuan, Pingtung 91201, Taiwan; 3 Department of Animal Science, National Pingtung University of Science and Technology, Pingtung 91201, Taiwan; 4 Department of Animal Science, National Chung Hsing University, Taichung 40227, Taiwan; 5 The iEGG and Animal Biotechnology Center, National Chung Hsing University, Taichung 40227, Taiwan

**Keywords:** Line, Floor Material, Feather Characteristics, Growth Performance, White Roman Geese

## Abstract

**Objective:**

The purpose of this study was to investigate whether goose growth and feather characteristics are influenced by their line and feeding surroundings, inclusive of floor materials and types, since there are no reports regarding these factors.

**Methods:**

The 240 White Roman geese which were hatched and sex identified came from 3 commercial goose farms. They were randomly distributed to 24 pens depending on a completely random design. The study continued for 13 weeks and included 3 lines of commercial geese and 2 floor types (cement strip floor [CSF] or cement floor [CF]).

**Results:**

The day one gosling weight from A farm was lower than other two farms (96 g vs 107 and 115 g; p<0.001). Afterwards, the body weight, back length, keel length, chest girth and main wing feather length among 3 farms showed no significance difference prior to 12 weeks. The CF group showed heavier body weight, shorter back length, longer keel length, shorter chest girth and shorter main wing feather length than the CSF group prior to 12 weeks. The down weight in the CF was heavier than the CSF group (57.1 g vs 41.8 g; p<0.01) prior to 13 weeks.

**Conclusion:**

The body weight showed the positive relations for dry feather weight (r = 0.59), down weight (r = 0.69), percent of the down weight of live body weight prior to 13 weeks (r = 0.61).

## INTRODUCTION

In accordance with the statistics of the Food and Agriculture Organization of the United Nations, the combined number of geese and guinea fowls in the world is 3.71 billion [1] and China’s share is 88.8%. According to the 2017 Agricultural Statistics Annual Report, geese numbers on the farm at the end of the year was 862 thousand, the value of raising geese was 15.4 billion TWD, the annual slaughter of geese reached 2.38 million or 0.28% of the total livestock production value in Taiwan.

Broilers raised on plastic floors are able to improve the air quality and cleanliness under a heat stress scenario [2]. On the contrary, they could be more susceptible to develop lesions in the breast, hock, and footpad. The different types of floor designs were on antimicrobial resistance in symbiotic *Escherichia coli* (*E. coli*) [3]. Hence, the floor type used in poultry management is able to reduce heat stress and bacterial contamination. Liu et al [4] indicated that the geese from wire floor during 1 to 28 days of age and then move to the floor during 29 to 70 days of age had higher feed conversion ratio than those systems. Then, Geese reared of wire floor had greater on body weight and body weight gain, otherwise, which had lower feed conversion ratio than those of systems.

The down processing industry has a history of more than 100 years, since the 19th century. With the greater consumer demand in recent years, the development of the down market has increased, and the down processing industry has also developed rapidly. Goose down is often used in high-price clothing and bedding. Feathers are a by-product of the meat industry that are divided into three categories: feathers, semi-feathers and down feathers; goose feathers weigh about 5% to 7% of live weight [5,6]. Each goose’s feathers weigh about 250 to 300 g and down feathers weigh about 20% of the total feathers and weigh about 50 to 60 g [7]. The effects of feather development, including nutrient, management, humidity, stock density, ventilation, etc., in geese. However, the heritability of the feather production capability is relatively low (h^2^ = 0.35) [8]. For feather growth, an unfavourable factor remained more than 70% of relative humidity. Geese reared on a wire floor, the body weight and feed conversion ratio were better than for other groups (floor, floor to free range) of Yangzhou geese [4]. The primary feather lengths in the feeding space of 2 birds/m^2^ had reduced 20.38% and 6.62% than that of 6 birds/m^2^ at 42 d and 70 d of geese, respectively [9].

Geese at 10 weeks age had heavier feathers than those at 12 weeks, while geese at 12 weeks produced more down [10]. The mean daily change in primary feather length was 2.6%, which is consistent with rates reported for other waterfowl species [11]. Furthermore, after slaughtering, 90 to 220 g marketable feathers can be obtained per goose from 9 to 30 weeks of age [6]. The quantity of raw feather and percentage of down shared the correlation [12].

In summary, high stocking densities result in increased feather foraging, as well as poor feathers and walking ability, which do not satisfy the welfare of the goose and may reduce the quality of the goose products [13]. In addition, on the free-range farm, the condition of feathers is significantly better than that in the stable goose farms [14]. At present, the feather raw materials in Taiwan, except for imports, are purchased from the slaughterhouse. In Taiwan, Animal welfare is highly valued, and techniques such as live feathering are prohibited. Generally, goose feathers or down are taken after slaughter, and subsequent cleaning and processing are carried out [15]. The objective of this study was to clarify the effects of lines and floor type on growth performance and feather characterization during the growth period of White Roman geese.

## MATERIALS AND METHODS

### Animals and experimental design

The experimental protocol was approved by the Animal Care and Use Committee of Changhua Animal Propagation Station, Livestock Research Institute, Council of Agriculture, Executive Yuan, Taiwan (IACUC 10305). The 750 eggs employed in this study were collected from three commercial goose farms in Taiwan, respectively. The eggs were then placed into an automatic incubator (Hoong Sheng Incubators Co. Ltd., Taiwan) set at 37.7°C for days 0 to 14, at 37.5°C for days 15 to 28, and at 37.2°C during hatching. The transparency of the fertilized eggs was judged with the naked eye on the 7th day of incubation. Hatchability was defined as the percentage of goslings hatched over the number of total eggs incubated.

The experimental animals were 240 White Roman geese from 3 commercial goose farms which were hatched under gender identification. 80 goslings were randomly took from each farm. They were randomly distributed in 24 pens depending on a completely random design and fed a grower diet *ad libitum* during the growth period. At the beginning of the study, 3 species of commercial geese and 2 floor type (cement strip floor [CSF] or cement floor [CF]) treatments lasted for 13 weeks (from hatch to 13 weeks), respectively. In this study, therefore, 3×2 factorial experiments were arranged.

### Feeding management

During the experimental period, the goslings were given 24-hour light from 0 to 4 weeks. Then, natural light was given during the growth period. The feed and drinking water were fed *ad libitum* during the experimental period. The geese were fed a gosling diet containing 20% crude protein, 2,900 metabolizable energy kcal/kg during 0 to 4 weeks. The grower diet contained 15% crude protein and 2,750 metabolizable energy kcal/kg during 5 to 13 weeks (Table 1).

The brooding house was 1.92 m ^2^ per pen. The length and width of each pen in the house were 1.50 m and 1.28 m, respectively, during 0 to 2 weeks. The stocking density was 5.21 /m^2^, and comprehensive vitamins were added from hatching to the drinking water on the 4th day. After 2 weeks, the geese were moved to a high CSF or CF of the grower house and raised to 13 weeks. The length and width of each pen of the grower house are 3.95 m×2.50 m, and the pen was 9.88 m^2^. The number of geese raised in each pen was calculated, in which the stocking density was 1.01 /m^2^. There is a water bath in each pen, which was 0.50, 0.40, and 0.15 m, respectively. Each pen has one feeding tank and one automatic drinking water tank, which were cleaned twice a week.

### Growth performance

At hatch, 4, 8, 12, and 13 weeks, the performance of the geese was assessed by measuring the body weight. The back length, keel length and chest girth were measured at 4, 8, 12, and 13 weeks. Back length was measured from the first spine to the base of the tail. Keel length measured keel bone by the ruler. Chest girth measured the circumference of the anterior end of the keel bone by the ruler. The length of main wing feather was measured by the ruler at the 7th, 8th, and 9th of main wing feather.

### Feather characteristic

At 13 weeks old, a total of 12 geese (6 males and 6 females) were randomly selected (each treatment containing 2 males and 2 females) and then killed by exsanguination from each goose. The samples for the carcass characteristics and harvest per goose were weighed, individually.

After the geese were slaughtered, the feathers obtained after depilation are called total hair. The total feather is washed with water for one hour, and dried in a dryer at 120°C for 6 minutes. The dried feathers are placed in an oven at 105°C for drying. After air drying, the dried feathers were weighed and manually divided into 10 cm or more, 4 to 10 cm, and less than 4 cm, and then weighed, respectively.

After the feathers were cleaned. The feathers below 4 cm are weighed via the analysis of down feathers to obtain the down weight of each goose. The samples were thoroughly mixed and sampled. The delicate feather are taken after the impurities are processed by the sanding machine. From the delicate feathers, the test is carried out by the quartering method, taking 10 to 15 grams for assay. The delicate feather is divided into pompon down, small feather (below 6.5 cm): small feather, down the wire: down fiber, shell fiber: chicken fiber, impurity, residual matter, etc., and record its weight respectively [16].

### Statistical analysis

The data collected were statistically analyzed using general linear models procedure of SAS software [17] following a completely randomized design. Data on the line treatments and sex ratio were subjected to analysis of variance using Statistical Analysis System Institute Package (SAS) and the mean values were compared using the LSMEANS with the significant level at p<0.05.

The mathematic model was:

$${\text{Y}}_{\text{ijk}}=\mu +{\text{L}}_{\text{i}}+{\text{F}}_{\text{j}}+{\left(\text{L}\times \text{F}\right)}_{\text{ij}}+{ɛ}_{\text{ijk}}$$

where Y_ijk_ is the measurement on average of birds in pen k, pen given floor type treatment j and line treatment i; μ is the overall mean; L_i_ is the fixed effect of line treatment i; F_j_ is the fixed effect of floor type treatment j; (L×S)_ij_ is the two-way interaction of line i by floor type treatment j; ɛ_ijk_ is the residual term that ɛ_ijk_ ∩ N (0, σ^2^ɛ).

## RESULTS

### Line on growth performances

A comparison of the growth performance and feather characteristics of White Roman geese from the three geese farms (Table 2) was carried out. The day one gosling weight in A farm was lower than in other farms (96 g vs 107 and 115 g; p<0.001). Afterwards, the body weight, back length, keel length, chest girth and main wing feather length among farm treatments showed no significance at 12th and 13th weeks. Feed conversion ratio in the CSF group was better than the other CF group (3.79 vs 4.59; p<0.001) from hatching period to 13th week.

### Floor types on growth performance

The body weight in the CF group was heavier than the other CSF group (5.22 kg vs 4.82 kg; p<0.001) at 12 weeks. The back length in the CF group had shorter than the other CSF group (29.5 cm vs 30.4 cm; p<0.01) at 12 weeks. The keel length in the CF group was longer than the other CSF group (22.4 cm vs 18.1 cm; p<0.001) at 12-weeks. The chest girth in the CF group was shorter than the other CSF group (44.6 cm vs 47.4 cm; p<0.001) at 12-weeks. The main wing feather length in the CF group was shorter than the other CSF group (29.5 cm vs 31.8 cm; p<0.001) at 12 weeks. Feed conversion ratio in the CSF group was better than the other CF group (3.79 vs 4.59; p<0.001) during hatch to 13 week.

### Line and floor type on feather growth

The comparison of farm flock and floor type traits of White Roman goose is shown in Table 3. The feather weight and down weight and the percentage of down among the spice treatments show no significant difference. The body weight and feather weight in the CSF tended to be lighter than the other CF group (p<0.1) at 13 weeks. The down weight in the CF was heavier than the other CSF group (57.1 g vs 41.8 g; p<0.01) at 13 weeks. The percent of the down weight of the live body weight in the CF was heavier than the other CSF group (24.0% vs 20.0%; p<0.05) at 13 weeks. Less than 4 cm feather weight in the CF was heavier than the other CSF group (95.6 g vs 79.4 g; p<0.05) at 13 weeks.

### Correlation coefficients among growth and feather characteristics

The effect of correlation coefficients among growth and feather characteristics of in grower geese are shown in Table 4. The keel length at 12 weeks and body weight at 13 weeks (r = 0.64; p<0.001), dry feather weight (r = 0.52; p<0.001), down weight (r = 0.61; p<0.001) and the percent of down weight of live body weight (r = 0.55; p<0.001) had a significant positive correlation in White Roman geese. Then, with greater than 10 cm feather weight (r = −0.61; p<0.001), it had a significant negative correlation in grower geese. The body weight at 13 weeks and dry feather weight (r = 0.59; p<0.01), down weight (r = 0.69; p<0.001), per cent of down weight of live body weight (r = 0.61; p<0.001), and less than 4 cm feather weight (r = 0.66; p<0.001) had a significant positive correlation.

### Regressions of various variables measured in grower geese under week ago

Regressions were calculated for those variables that responded significantly to varying week age. If week age is defined as X (week/geese) and body weight is defined as Y1 (kg/bird), then Y_1_ = −1.21+0.90X–0.0322X^2^ (R^2^ = 0.922, p<0.0001) for the entire experimental period (Table 5). Stemum length is defined as Y2 (cm/bird), then Y_2_ = 28.8–9.68X+1.641X^2^ (R^2^ = 0.765, p = 0.0004). Chest girth is defined as Y3 (cm/bird), then Y_3_ = 15.0+3.98X–0.1148X^2^ (R^2^ = 0.937, p = 0.0016). Feather length is defined as Y5 (cm/bird), then Y_5_ = −46.6+17.2X–1.40 X^2^+0.0418 X^3^ (R^2^ = 0.986, p<0.0001) of whole period (Table 5).

## DISCUSSION

The body weight increases with age of domestic geese [18]. The Embden Goose had heavier carcass weight than Toulouse and its cross groups [19] and Egyptian Geese [20,21]. The Embden breed has a heavy body weight and faster gain weight. The gosling weight was 60.9% of egg weight before setting [22]. The lower weight of goslings (89.0 g) was lighter than the highest weight group (133.0 g) at 10 weeks. Then, the egg weight was significantly correlated with hatching weight (72%); significant regression was also found between egg weight and hatching weight (r^2^ = 0.51) [23]. The results of this study showed that the growth performance of the three goose farms had no significant difference (Table 2). Therefore, the body weight of day-one gosling in A farm was lower than those of their counterparts on the other farms. Afterwards, the body weight, back length, keel length, chest girth and main wing feather length among farm treatments had no significant difference at 12 weeks. The body weight of day-one gosling in A farm was lighter than among the other groups. The reason was that the eggs of A farm came from first parity, then the B and C farm were second parity. However, the correlation of egg weight to chick weight decreases with the increasing age of the chick [24]. That observation is in agreement with our results.

Meat quality was assessed in Cobb-500 cages and floor-fed broilers, and meat quality estimated by a set of related parameters. Broilers raised on a plastic floor showed better health scores and lower hock injury rates than the others raised in the wood shaving group [25]. Broilers raised on a plastic floor could improve air quality and cleanliness during heat stress [2]. Contrarily, they could be more susceptible to developing lesions in the breast, hock, and footpad. Since chickens are more susceptible to develop lesions on the carcass, being a source of pain, it impairs bird wellbeing and causing losses in meat production. Chuppava et al [3] evaluated the effect of different types of floor designs on antimicrobial resistance in symbiotic *E. coli* treated with enrofloxacin. The present study showed that the CF had heavier body weight, shorter back length, longer keel length, shorter chest girth and shorter main wing feather length than the other CSF group at 12 weeks (Table 2). Conversely, Farghly et al [26] indicated that using plastic and wood slatted floors could improve growth performance and meat quality with an increase of body weight, daily gain, feed conversion, tenderness and juiciness than cement, wire net and rubber mat groups of turkeys. This is probably on account of the fact that growing geese are only raised on the concrete floor (CF), which is easy to cause muddy on the ground. Although the weight of the 12-week-old body is significantly higher than that of the strip-like ground (CSF), it is disadvantageous to the shape of the feather growth period. The goose needs to be keep in the dry breeding environment and prevent from fecal contamination.

Many factors influence the feather quality of waterfowls, inclusive of nutrition, weather, housing, stock density, ventilation, and relative humidity, etc. Szado et al [27] indicated that 1.6% fat content in diets are capable of increasing 3.2% down in geese. Then, the authors show geese rearing on higher relative humidity is adversely affected. The feather quantity was evaluated by body weight in live geese [28]. The high stocking density will induce stress, which adversely on body weight and feathers, etc [9]. Based on other reports, male geese exhibited more feathers and down than females [12]. The average live weight of male and female geese at 16 weeks of age was 4,371 g and 4,071 g, respectively [29]. There is intensive Vexilla in the female because their barbs are thinner than those of the males [30]. The correlation between quantity and raw feather and percentage of down is significant, but the regression value in case of males is negative [12]. Down sampled from the third feather, harvesting is 0.136 g for the layer and 0.143 g for the ganders on average [31]. Moreover, it showed that the amount of down is related to the surface area of goose. Due to the weight and size of the male goose (Stemum length and Chest girth), the feathers of the male goose are mainly used for appearance and more protection. Male goose weighs more than the female goose, as reported in the past. The difference between the genders begins during the incubation period. More intensive vexilla are found in the female because their barbs which are thinner than those of the males and there is no gender difference in feather weight. That the mean daily change in primary feather length was 2.6%, which is consistent with rates reported in other waterfowl species [11]. That after slaughter, 90 to 220 g of marketable feathers could be obtained per goose from 9 to 30 weeks [6]. Furthermore, it has been reported that β-catenin signaling causes feather bud development. The results in this study showed that the feathers weight and down weight and the percentage of down among the spices treatments reveal no significant difference (Table 3). The body weight and feather weight in the CSF tended to be lighter than the other CF group at 13 weeks. The result implies a positive relationship between the feather weight and body weight. The result showed that significant regression between body weight and dry feather weight or down weight (r^2^ = 0.59 or 0.69, Table 4). However, the feather weight was heavier in young geese at 10 weeks, and down weight was greater in the older group at 12 weeks [10]. It showed down had not reached maturity in young geese. The difference in live weight between the genders was significant during 6 to 16 weeks [29]. Therefore, it suggested that CFs could enhance the growth performance, feather and down weight. The discrepancy between our results and those of author [32] may be caused by differences in breed, season, and experimental conditions applied. The results in this study show that the body weight with dry feather weight and down weight had a positive correlation (Table 4). That heavier live weight, longer neck trunk and deeper chest in male native Turkish geese might cause these differences in feather and down production [10].

## CONCLUSION

Our results suggested that the CF group shared heavier body weight than the other CSF group prior to 12 weeks. The down weight in the CF shared heavier than the other CSF group. Then, body weight shared the positive relations for dry feather weight, down weight, percent of the down weight of live body weight prior to 13 weeks.

## Figures and Tables

**Table 1:** The components of basal diet of White Roman geese

Items	Basal diet	

	Starter (0 to 4 wk)	Grower (5 to 13 wk)
Ingredients (kg/ton)		
Yellow corn, ground	610.5	670.5
Soybean meal	260	165
Wheat bran	20	50
Fish meal, 65%	50	25
Molasses	30	30
Salt	3	3
Dicalcium phosphate	10	13
Limestone, pulverized	7	7
Choline chloride, 50%	1	1
DL-methionine	2.5	2
Rice bran	-	30
Vitamin premix1)	4	2
Mineral premix2)	2	1.5
Total	1,000	1,000
Calculated values		
Crude protein (%)	20	15
Metabolizble energy (kcal/kg)	2,900	2,750

1) Vitamin premix: Each kg containing vitamin A 10,000,000 IU, vitamin D_3_ 2,000,000 IU, vitamin E 20,000 IU, vitamin B_1_ 1 g, vitamin B_2_ 4.8 g, vitamin B_6_ 3 g, vitamin B_12_ 0.01 g, biotin 0.2 g, vitamin K_3_ 1.5 g, D-calcium pantothenate 10 g, folic acid 0.5 g, nicotinic acid 25 g.

2) Mineral premix: Each kg containing Cu 15.0 g, Fe 80 g, Zn 50 g, Mn 80 g, Co 0.25 g, I 0.85 g.

**Table 2:** A comparison of the growth performance of White Roman geese from the three geese farms

Items	Line			SEM	Floor type		SEM	Significance		
		
	A	B	C		CSF	CF		F	FT	F×FT
Body weight (kg/goose)										
0 wk	0.096^c^	0.107^b^	0.115^a^	0.001	0.106	0.106	0.001	***	NS	NS
4 wk	1.88	1.92	1.85	0.023	1.89	1.89	0.018	†	NS	NS
8 wk	4.09	4.10	3.99	0.053	4.03	4.04	0.042	NS	NS	NS
12 wk	4.89	5.01	5.12	0.150	4.82^y^	5.22^x^	0.122	NS	***	NS
13 wk	5.00	5.08	5.48	0.233	4.89	5.48	0.191	NS	†	NS
Body weight gain per day (kg/bird/d)										
Hatch-13 wk	0.05	0.05	0.06	0.003	0.05	0.06	0.002	NS	†	NS
Feed conversion ratio (kg feed/kg gain)										
Hatch-13 wk	3.96	4.41	4.20	0.179	4.59^x^	3.79^y^	0.147	NS	***	NS
Back length (cm/bird)										
4 wk	22.2^ab^	22.9^a^	21.8^b^	0.184	22.3	22.3	0.143	***	NS	**
8 wk	28.7^a^	28.5^ab^	27.9^b^	0.271	28.8^x^	27.9^y^	0.213	*	**	NS
12 wk	30.0	30.0	29.7	0.279	30.4^x^	29.5^y^	0.219	NS	**	NS
13 wk	30.0	30.2	31.4	0.382	30.6	30.3	0.312	NS	†	NS
Stemum length (cm/bird)										
4 wk	11.5	11.5	11.5	0.114	11.5	11.5	0.089	NS	NS	NS
8 wk	18.2	18.0	18.3	0.175	16.8^y^	19.6^x^	0.137	NS	***	NS
12 wk	20.1	20.4	20.2	0.186	18.1^y^	22.4^x^	0.147	NS	***	NS
13 wk	20.3	20.1	20.7	0.367	17.4	17.4	0.300	NS	NS	NS
Chest girth (cm/bird)										
4 wk	28.5^b^	29.8^a^	29.0^b^	0.242	29.1	29.1	0.188	***	NS	*
8 wk	40.2	40.3	40.1	0.313	42.1^x^	38.2^y^	0.246	NS	***	NS
12 wk	45.7	46.3	45.8	0.289	47.4^x^	44.6^y^	0.227	NS	***	†
13 wk	46.8	46.8	49.0	1.021	47.1	47.8	0.833	NS	NS	NS
Feather length (cm)										
4 wk	2.03	2.32	2.15	0.123	2.15	2.18	0.099	NS	NS	NS
8 wk	22.5	22.7	22.3	0.186	23.0	21.9	0.146	NS	***	*
12 wk	30.6	30.6	30.8	0.363	31.8^x^	29.5^y^	0.287	NS	***	NS
13 wk	32.5	30.6	32.0	0.840	32.6	31.0	0.686	NS	NS	NS

SEM, standard error of the mean; CSF, cement strip floor; CF, cement floor; F, flock; FT, floor type.

a–c Means within the same row under flock without the same superscripts differ significantly (p<0.05).

x,y Means within the same row under floor type without the same superscripts differ significantly (p<0.05).

NS, not significantly different;

† p<0.1;

* p<0.05;

** p<0.01;

*** p<0.001.

**Table 3:** A comparison of the feather characteristics of White Roman geese from the three geese farms

Items	Line			SEM	Floor type		SEM	Significance		
		
	A	B	C		CSF	CF		F	FT	F×FT
Body weight at 13 wk (kg/goose)	5.00	5.08	5.49	0.233	4.89	5.48	0.191	NS	†	NS
Feather weight (g/goose)	215	219	235	10.51	208	238	8.589	NS	†	NS
Feather weight (% of FDBW)	4.29	4.30	4.33	0.227	4.26	4.35	0.185	NS	NS	NS
Down weight (g/goose)	47.3	46.8	54.1	2.591	41.8^y^	57.1^x^	2.115	NS	**	*
Down weight (% of Feather weight)	22.0	21.1	22.9	1.004	20.0^y^	24.0^x^	0.820	NS	*	*
>10 cm feather weight (g/bird)	70.9	72.9	74.3	4.896	74.7	70.7	3.998	NS	NS	NS
4–10 cm feather weight (g/bird)	57.0	59.3	65.4	7.524	52.7	68.4	6.144	NS	NS	NS
<4 cm feather weight (g/bird)	86.2	84.9	91.5	5.436	79.4^y^	95.6^x^	4.439	NS	*	NS

SEM, standard error of the mean; CSF, cement strip floor; CF, cement floor; F, flock; FT, floor type; FDBW, eighteen-hour feed-deprived body weight.

x,y Means within the same row under floor type without the same superscripts differ significantly (p<0.05).

NS, not significantly different;

† p<0.1;

* p<0.05;

** p<0.01;

*** p<0.001.

**Table 4:** Partial correlation coefficients among growth, carcass and feather characteristics in grower geese

Item	BW 4	back4	t4	CG 4	BW 8	BL 8	SL 8	CG 8	BW 12	BL 12	SL 12	CG 12	FL4	FL 8	FL 12	BW 13	DFW	PDFW	DW	DOWP	L10CFW	L410CFW	L4CFW
BW 0	0.21***	0.14**	0.03	0.002	0.15**	0.09	0.04	0.05	0.02	0.06	−0.01	0.05	0.01	0.06	−0.04	0.14**	−0.09	−0.25***	−0.11*	−0.10*	0.18***	−0.14**	0.01
BW 4		0.29***	0.33***	0.36***	0.58***	0.14**	0.22***	0.27***	0.25***	0.10	0.13*	0.23***	0.32***	0.37***	0.05	−0.03	−0.09	−0.06	−0.04	0.00	0.07	−0.13*	−0.01
BL 4			−0.23***	0.52***	0.24***	0.56***	−0.14*	0.29***	−0.52***	0.54***	−0.11*	0.43***	−0.19***	−0.05	−0.16**	−0.06	−0.17**	−0.14*	−0.08	−0.01	0.08	−0.23***	−0.04
SL 4				−0.30***	−0.02	−0.59***	0.34***	−0.23***	0.71***	−0.62***	0.31***	−0.37***	0.66***	0.40***	0.13*	0.05	0.03	−0.01	0.04	0.04	−0.03	0.02	0.03
CG 4					0.35***	0.57***	−0.14*	0.37***	−0.39***	0.55***	−0.16**	0.51***	−0.24***	−0.08	−0.02	−0.07	−0.13*	−0.09	−0.04	0.02	0.00	−0.15**	−0.04
BW 8						0.45***	0.18***	0.46***	0.20***	0.43***	0.13*	0.52***	−0.09	0.19***	0.24***	−0.01	−0.06	−0.05	0.02	0.06	0.04	−0.14*	0.03
BL 8							−0.32***	0.56***	−0.64***	0.82***	−0.29***	0.69***	−0.58***	−0.16**	−0.04	−0.13*	−0.15**	−0.04	−0.14**	−0.10	0.23***	−0.18***	−0.09
SL 8								−0.34***	0.52***	−0.32***	0.68***	−0.37***	0.32***	0.08	−0.07	0.53***	0.49***	0.07	0.53***	0.45***	−0.54***	0.35***	0.50
CG 8									−0.25***	0.55***	−0.45***	0.70***	−0.27***	0.17**	0.29***	−0.47***	−0.42***	−0.03	−0.45***	−0.38***	0.46***	−0.30	−0.43
BW 12										−0.65***	0.44***	−0.35***	0.60***	0.34***	0.27***	0.15**	0.12*	−0.01	0.15**	0.14*	−0.15**	0.08	0.12
BL 12											−0.31***	0.73***	−0.62***	−0.17**	0.07	−0.19***	−0.16**	0.01	−0.17**	−0.14*	0.18*	−0.13	−0.14
SL 12												−0.40***	0.28***	−0.02	−0.16*	0.64***	0.52***	0.01	0.61***	0.55***	−0.61***	0.32***	0.59***
CG 12													−0.39***	0.09	0.28***	−0.39***	−0.38***	−0.05	−0.38***	−0.30***	0.43***	−0.32***	−0.35***
FL 4														0.56***	0.06	0.08	−0.04	−0.12*	−0.03	−0.02	0.01	−0.02	−0.04
FL 8															0.43***	−0.14*	−0.23***	−0.15**	−0.26***	−0.23***	0.21***	−0.12*	−0.26***
FL 12																−0.24***	−0.22***	−0.03	−0.23***	−0.20***	0.18***	−0.12*	−0.24***
BW 13																	0.59***	−0.29***	0.69***	0.61**	−0.59***	0.34***	0.66***
DFW																		0.60***	0.84***	0.63***	−0.54***	0.75***	0.81***
PDFW																			0.33***	0.18***	−0.05	0.53***	0.34***
DW																				0.95***	−0.68***	0.36***	0.96***
DOWP																					−0.61***	0.07	0.93***
L10CFW																						−0.52***	−0.54***
L410CFW																							0.26***

BW, body weight (kg/bird); BL, back length; SL, stemum length (cm/bird); CG, chest girth (cm/bird); DFW, dry feather weight; PDFW, dry feather weight (% of body weight); DW, down weight; DOWP, down (% of dry feather weight); L10CFW, >10 cm feather weight (g/bird); L410CFW, 4–10 cm feather weight (g/bird); L4CFW, <4 cm feather weight (g/bird).

* p<0.05;

** p<0.01;

*** p<0.001.

**Table 5:** The regressions of various variables measured in grower geese under week ago (X, age of week/geese)

Item	Regression equation	p-value
Body weight (Y_1_ kg/bird)	Y_1_ = −1.21+0.90X-0.0322X^2^	<0.0001
Stemum length (Y_2_ cm/bird)	Y_2_ = 28.8–9.68X+1.641X^2^	0.0004
Chest girth (Y_3_ cm/bird)	Y_3_ = 15.0+3.98X–0.1148X^2^	0.0016
Back length (Y_4_ cm/bird)	Y_4_ = 14.8+2.41X–0.0943X^2^	<0.0001
Feather length (Y_5_ cm/bird)	Y_5_ = −46.6+17.2X–1.40 X^2^+0.0418 X^3^	0.0159

## References

[1] FAO (2017). FAOSTAT Database Results.

[2] de Almeida EA, Sant’anna AC, Crowe TG, Macari M, Furlan RL (2018). Poultry rearing on perforated plastic floors and the effect on air quality, growth performance, and carcass injuries - Experiment 2: Heat stress situation. Poult Sci.

[3] Chuppava B, Keller B, Meißner J, Kietzmann M, Visscher C (2018). Effects of different types of flooring design on the development of antimicrobial resistance in commensal *Escherichia coli* in fattening turkeys. Vet Microbiol.

[4] Liu BY, Wang ZY, Yang HM (2011). Influence of rearing system on growth performance, carcass traits, and meat quality of Yangzhou geese. Poult Sci.

[5] Del Hoyo J, Elliott A, Sargatal J (1992). Handbook of the birds of the world. Vol. l. Ostrich to Ducks.

[6] Szigeti M (1987). Poultry waste utilization in poultry industry. Baromfitenyésztésés Feldolgozás.

[7] Schneider KH (1995). Geese - A guide to their breeding, keeping, feeding.

[8] Kozák J (2011). An overview of feathers formation, moults and down production in geese. Asian-Australas J Anim Sci.

[9] Yin LY, Wang ZY, Yang HM, Xu L, Zhang J, Xing H (2017). Effects of stocking density on growth performance, feather growth, intestinal development, and serum parameters of geese. Poult Sci.

[10] Saatci M (2008). Effects of age, sex, feather colour, body measurements, and body weight on down and feather yield in Native Turkish geese. Turk J Vet Anim Sci.

[11] Van de Wetering D, Cooke F (2000). Body weight and feather growth of male barrow’s goldeneye during wing molt. Condor.

[12] Kozák J, Bodi L, Karsai KM, Monostori K The effect of sex on the feather production of geese.

[13] Yin L, Yang H, Xu L, Zhang J, Xing H, Wang Z (2017). Feather performance, walking ability, and behavioral changes of geese in response to different stocking densities. Appl Anim Behav Sci.

[14] Mehmet AB, Sarica M, Yamak US (2017). Effect of production system on foot pad dermatitis (FPD) and plumage quality of geese. Eur Poult Sci.

[15] Kozák J, Gara I, Kawada T (2010). Production and welfare aspects of goose down and feather harvesting. World’s Poult Sci.

[16] Japanese Standards Association (2011). JIS L 1903: 2011 Testing methods for feathers.

[17] SAS Institute (2014). Sas/Stat Guide for Personal Computers. Version 9. 01.

[18] Hamadani H, Khan AA, Ganai TAS, Banday MT, Hamadani A (2014). Growth and production traits of domestic geese in local conditions of Kashmir, India. Indian J Anim Sci.

[19] Solé M, Peña F, Domenech V (2016). Carcass and meat quality traits in an embden×toulouse goose cross raised in organic *Dehesa*. Asian-Australas J Anim Sci.

[20] El-Hanoun AM, Attia YA, Gad HAM, Abdella MM (2012). Effect of different managerial systems on productive and reproductive traits, blood plasma hormones and biochemical constituents of geese. Animal.

[21] El-Hanoun AM, Attia YA, AL-Harthi MA, Habiba HI, Oliveira MC (2017). Magnetized drinking water improves productivity and blood parameters in geese. Rev Colomb Cienc Pecu.

[22] Kucharska-Gaca J, Adamski M, Kuźniacka J, Kowalska E (2016). Influence of the weight of hatching eggs on the hatchability indices and on the body weight of geese in rearing and after fattening with oats. Acta Sci Pol Zootechnica.

[23] Saatci M, Kirmizibayrak T, Aksoy AR, Tilki M (2005). Egg weight, shape index and hatching weight and interrelationships among these traits in native Turkish Geese with different coloured feathers. Turk J Vet Anim Sci.

[24] Wilson HR (1991). Interrelationships of egg size, chick size, posthatching growth and hatchability. World’s Poult Sci.

[25] Fisinin VI, Lukashenko VS, Saleyeva IP (2018). Meat quality in broilers reared in different housing systems. Voprosy Pitaniia.

[26] Farghly MFA, Mahrose KM, Cooper RG, Ullah Z, Rehman Z, Ding C (2018). Sustainable floor type for managing turkey production in a hot climate. Poult Sci.

[27] Szado J, Pakulska E, Kapkowska E Influence of production factors on feather quality.

[28] Tóth S, Szélné Szeri M, Nguyen DV (1988). Factors affecting feather production of geese. Állattenyésztés és Takarmányozás.

[29] Tilki M, Saatci M, Kirmizibayrak T, Aksoy A (2005). Effect of age on growth and carcass composition of Native Turkish Geese. Archiv fur Geflugelkunde.

[30] Yiliz D, Bozkurt EU, Akturks SH (2009). Determination of goose feather morphology by using SEM. J Anim Vet Adv.

[31] Kozák J (1999). Poultry Industry of Hungary and its EU-conform regulation. Technologies, market regulation and animal welfare.

[32] Fraley SM, Fraley GS, Karcher DM, Makagon MM, Lilburn MS (2013). Influence of plastic slatted floors compared with pine shaving litter on Pekin Duck condition during the summer months. Poult Sci.

